# The burden of prostate cancer in Trinidad and Tobago: one of the highest mortality rates in the world

**DOI:** 10.1007/s10552-018-1038-8

**Published:** 2018-05-17

**Authors:** Wayne A. Warner, Tammy Y. Lee, Fang Fang, Adana A. M. Llanos, Smriti Bajracharya, Vasavi Sundaram, Kimberly Badal, Vandana Devika Sookdeo, Veronica Roach, Marjorie Lamont-Greene, Camille Ragin, Simeon Slovacek, Krishan Ramsoobhag, Jasmine Brown, Timothy R. Rebbeck, Ravi Maharaj, Bettina F. Drake

**Affiliations:** 1 Oncology Division, Siteman Cancer Center, Department of Cell Biology and Physiology, Washington University School of Medicine, St. Louis, MO USA; 2 MedSeq HealthCare Solutions, Trincity, Trinidad and Tobago; 3 A Fighting Chance, Trincity, Trinidad and Tobago; 4 California State University, Los Angeles, CA USA; 5 Division of Public Health Sciences, Washington University School of Medicine, 600 S. Taylor Ave., Campus Box 8100, St. Louis, MO 63110 USA; 6 Rutgers School of Public Health and Rutgers Cancer Institute of New Jersey, Rutgers University, New Brunswick, NJ USA; 7 Center for Public Health Systems Science, George Warren Brown School of Social Work, Washington University, St. Louis, MO USA; 8 Department of Genetics, Center for Genome Sciences and Systems Biology, Washington University School of Medicine, St. Louis, MO USA; 9 Caribbean Cancer Research Initiative, San Fernando, Trinidad and Tobago; 10 Department of Clinical Surgical Sciences, Faculty of Medical Sciences, University of the West Indies, St. Augustine, Trinidad and Tobago; 11 Dr. Elizabeth Quamina Cancer Registry, Eric Williams Medical Sciences Complex, Mt. Hope, Trinidad and Tobago; 12 Cancer Prevention and Control Program, Fox Chase Cancer Center-Temple Health, Philadelphia, PA USA; 13 African-Caribbean Cancer Consortium, Philadelphia, PA USA; 14 Department of Otolaryngology - Head and Neck Surgery, Temple University School of Medicine, Philadelphia, PA USA; 15 College of Public Health, Temple University, Philadelphia, PA USA; 16 Urology Department, San Fernando General Hospital, San Fernando, Trinidad and Tobago; 17 Washington University, St. Louis, MO USA; 18 Department of Medical Oncology, Dana Farber Cancer Institute, Boston, MA USA; 19 Department of Epidemiology, Harvard TH Chan School of Public Health, Boston, MA USA; 20 Alvin J. Siteman Cancer Center, St. Louis, MO USA

**Keywords:** Cancer in populations of African ancestry, Trinidad and Tobago, Prostate cancer, Caribbean, Geography, Cancer incidence, Cancer mortality, Cancer survival

## Abstract

**Purpose:**

In Trinidad and Tobago (TT), prostate cancer (CaP) is the most commonly diagnosed malignancy and the leading cause of cancer deaths among men. TT currently has one of the highest CaP mortality rates in the world.

**Methods:**

6,064 incident and 3,704 mortality cases of CaP occurring in TT from January 1995 to 31 December 2009 reported to the Dr. Elizabeth Quamina Cancer population-based cancer registry for TT, were analyzed to examine CaP survival, incidence, and mortality rates and trends by ancestry and geography.

**Results:**

The age-standardized CaP incidence and mortality rates (per 100,000) based on the 1960 world-standardized in 2009 were 64.2 and 47.1 per 100,000. The mortality rate in TT increased between 1995 (37.9 per 100,000) and 2009 (79.4 per 100,000), while the rate in the US decreased from 37.3 per 100,000 to 22.1 per 100,000 over the same period. Fewer African ancestry patients received treatment relative to those of Indian and mixed ancestry (45.7%, 60.3%, and 60.9%, respectively).

**Conclusions:**

Notwithstanding the limitations surrounding data quality, our findings highlight the increasing burden of CaP in TT and the need for improved surveillance and standard of care. Our findings highlight the need for optimized models to project cancer rates in developing countries like TT. This study also provides the rationale for targeted screening and optimized treatment for CaP to ameliorate the rates we report.

**Electronic supplementary material:**

The online version of this article (10.1007/s10552-018-1038-8) contains supplementary material, which is available to authorized users.

## Introduction

With an estimated 1.1 million annual cases diagnosed globally in 2012, prostate cancer (CaP) is the second most prevalent cancer in men accounting for 15% of the cancers diagnosed [1]. Overall, it is the fourth most prevalent cancer among men and women and the fifth leading cause of male cancer mortality [1]. In parallel with the rate of adoption of prostate-specific antigen (PSA) testing and subsequent biopsy, there is a 25-fold variation in CaP incidence rates globally. Rates are highest in developed countries (e.g., Australia/New Zealand and Northern America, age-standardized rate (ASR) 111.6 and 97.2 per 100,000, respectively), and in less developed regions such as the Caribbean (79.8 per 100,000) relative to South-Central Asia (4.5 per 100,000) where the rates are lowest [1]. Mortality rates are generally high in regions with high populations of African descent (Caribbean, 29–55 per 100,000 and Sub-Saharan Africa, ASRs 19–24 per 100,000), and very low in South-Central Asia (2.9 per 100,000) [1].

As in most Caribbean nations, non-communicable diseases (NCDs) including diabetes, cardiovascular disease, and cancers represent the largest causes of morbidity and mortality. The population of Trinidad and Tobago (TT) in 2010 was 1,328,019 with a diversity of ancestral groups including African (34.2%), East Indian (35.4%), mixed (22.8%), unknown (6.2%), and all other ethnic groups (Chinese, White, and Syrian/Lebanese) totaling 1.4% [2]. With its petrochemical resources, it is classified as a high-income country by the World Bank [3], yet a developing country by the International Monetary Fund (IMF) [4], and is a member of the United Nations Conference of Small Island Developing States (SIDS) [5].

TT has a bipartite healthcare system with a public access system offering free prescription drugs and other pharmaceutical items to all citizens as well as a private system where medical services are covered to varying degrees by the patient’s private insurance. The Ministry of Health provides overall healthcare goals and strategic policy to the five Regional Health Authorities (RHAs) that manage the public health facilities. Each RHA manages public health facilities which provide medical services within their catchment areas. These services are provided at a ground-level primary care approach with local health centers (LHC) (86 in Trinidad and 19 in Tobago) and a specialized approach with multi-specialty serviced hospitals [6]. TT has five RHAs which collectively manage four general hospitals, two district hospitals, and four specialist hospitals (psychiatric, maternity, thoracic, and a combined radiotherapy/physical medicine/gerontology facility) [7].

The epidemiological landscape of prostate cancer in TT has not been described, despite reports that the CaP mortality rate in TT is one of the highest in the world [8–10]. Given its demographic profile and development status, TT presents a unique opportunity to analyze CaP incidence and mortality rates and trends relative to more developed countries. We present incidence and mortality rates and temporal trends for TT from 1995 to 2009 using the most up-to-date data available from the population-based, National Cancer Registry of TT (The Dr. Elizabeth Quamina Cancer Registry). We discuss disparities by ancestry, age, treatment, and geography and their potential impact on survival trends.

## Materials and methods

**Fig. 1:**
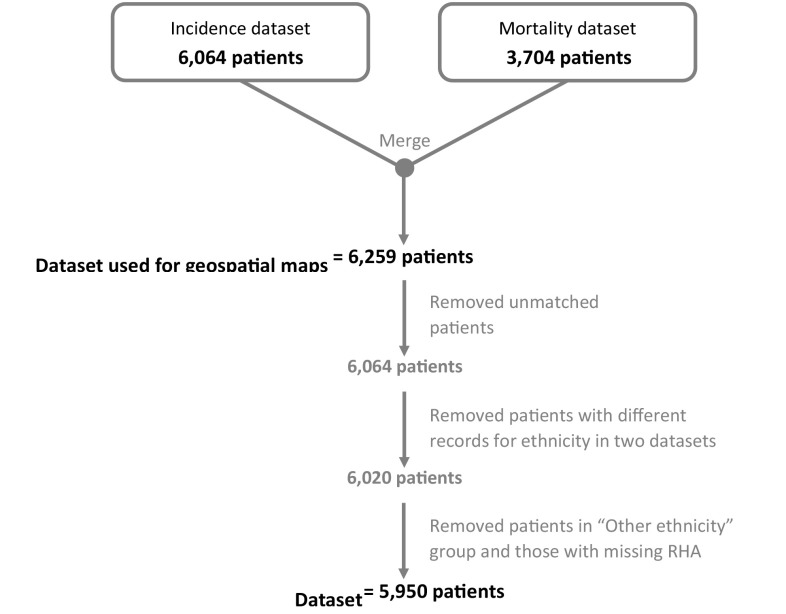
Patient selection algorithm

The National Cancer Registry of TT provided de-identified cancer surveillance data on 6,064 incident and 3,704 mortality cases of CaP diagnosed in TT from 1 January 1995 to 31 December 2009. This population-based cancer registry was established in 1994 by the Trinidad and Tobago Cancer Society, using cancer registry frameworks and policies set by the International Agency for Research on Cancer (IARC) [11, 12]. The registry uses the CanReg database and statistical software (version 4.33) to capture incidence and mortality data. The source of the cancer registry records was previously described [13]. In brief, the registry abstracts cancer surveillance data from both public and private medical institutions across Trinidad and Tobago including all of the main public cancer treatment centers. Abstracted data included patients’ home city, age, gender, ancestry, stage, grade, and method of cancer detection. All CaP cases were classified according to the International Classification of Diseases for Oncology (ICD-O) code C61.9 [14]. The record selection algorithm is shown in Fig. 1. The incidence and mortality cases were merged based on the patient’s registry code, yielding 6,259 records. Three hundred and nine records were excluded from the merged datasets resulting in an analytic dataset of 5,950 CaP cases. This study received IRB approval from the Institutional Review Boards of all participating institutions.

The boundaries for the geographic analysis by corporation and Regional Health Authority (RHA) were previously described [13]. The TT Central Statistical Office (CSO) provided the death certification and population data through the 2000 and 2010 census. The population pyramids for 2000 and 2011 were previously described [15, 16]. The CSO collects several population measures including age (single year of age, 5- and 10-year age groups), ethnicity, and gender. Population estimates for the other study years were calculated through interpolation using the “irregular points of year” estimation method [11, 12].

This cancer registry is the most reliable source of cancer surveillance data for the population of TT. The registry collects cancer surveillance records from public and private health institutions. Public sector reporting health institutions include Port of Spain General Hospital, Caura Hospital, National Radiotherapy Centre, Sangre Grande Hospital, Tobago Regional Hospital, Mount Hope Women’s Hospital, Eric Williams Medical Sciences Complex, San Fernando Hospital, and Point Fortin Area Hospital. Private sector reporting health institutions include Augustus Long Hospital, Petrotrin-Santa Flora Medical Center, Community Hospital of the Seventh-Day Adventists, Brian Lara Treatment Centre, and West Shore Private Hospital.

Imputation by binary logistic regression methodologies were used to ascribe ancestry among CaP cancer cases with unclassified ancestry [13]. In brief, four separate binary logistic regression models were calculated with four different ancestral groups which were by far the most prevalent in the census data. These were then used as the dependent variables (“African,” “Indian,” “Mixed,” and “Other: Chinese, White, Syrian/Lebanese”) for estimation purposes. Known demographic variables including such factors as gender, age, and corporation of residence were used as independent variables in the four predictive regression models to assign ancestry based on profiles of those cases with available race/ethnicity data. After fitting the four binary logistic regression models for the cases with missing race/ethnicity data, probabilities of predicted values of the dependent variables (ancestry) were calculated. These values showed the probabilities of each cancer patient falling into each of the four ancestral groups. Cancer patients with unknown race/ethnicity were then assigned to one of the four ancestral groups according to the highest probability of being a member of that group. From the incidence dataset, 1,620 (26.7%) cases were imputed with 1,481 and 139 cases imputed to African and Indian ancestry, respectively. For the mortality dataset, 2,132 cases were imputed, with 1,090 and 42 cases imputed to African and Indian ancestry, respectively.

### Statistical analysis

The statistical analysis methods were previously described [13]. In brief, we calculated the age-standardized incidence and mortality rates (per 100,000) based on the 1960 world-standardized [17] and the 2000 United States (US) standard population to compare TT CaP rates to global and US (Surveillance, Epidemiology, and End Results (SEER) Program) rates and trends, respectively. This methodology was selected to allow for comparison with the IARC data which use the same standardization. The NCI SEER*Stat software (version 8.2.1) was used to calculate the 2000 US age-standardized incidence and mortality rates [18] for comparisons to the US data. TT mortality data from the World Health Organization (WHO) Cancer Mortality Database for the study period [19] were compared to the results obtained from analyses based on the TT Cancer Registry data. GraphPad^®^ Prism (GraphPad Software, Inc) was used to create the survival curve and compare survival rates across regions. It uses the method of Kaplan and Meier and calculates the 95% confidence interval for fractional survival at any particular time.

### The mortality-to-incidence ratio

The mortality-to-incidence ratio (M/I) was calculated using the National Cancer Registry of TT dataset for 1995–2009. For comparison, M/I ratios were also calculated using incidence data from GLOBOCAN 2012 and mortality data from the WHO Mortality Database for 2012 [19, 20]. The sources and quality of these data have been previously described [1, 21]. The two countries in each WHO region with the maximal and minimum M/I ratios were included in our analysis as well as countries with similar ancestral distributions to the TT population. M/I ratios have a maximal value of 1.00, which is an indicator of poor survival.

#### Geospatial mapping

The geospatial maps were rendered in the R computing environment [22] using the ggplot command. Locations for Trinidad and Tobago were obtained by downloading a shapefile from http://www.gadm.org/country and plotted with the mortality and incidence rates as the fill color.

## Results

### Prostate cancer statistics

Our analytic dataset consisted of 5,950 CaP cases (78.4% of African ancestry, 12.1% of Indian ancestry, and 9.5% of mixed ancestry) recorded by the TT Cancer Registry for the period between 1 January 1995 and 31 December 2009. In TT, prostate cancer accounted for 41.7% of all new cancers and 37.4% of all cancer deaths among men (unpublished data). Table 1 shows the demographic and clinical characteristics of these incident CaP cases. The mean age at diagnosis was 72.6 years, with significant differences by ancestry. Specifically, CaP patients of Indian ancestry presented at a younger age (70.2 years), compared to those of mixed (72.3 years) or African ancestry (73 years).

**Table 1:** Characteristics of prostate cancer incident cases reported to the TT Cancer Registry, overall and by ethnicity, 1995–2009

Characteristics	Overall	African	Indian	Mixed
	Count (%)	Count (%)	Count (%)	Count (%)
Total number of prostate cancer cases	5,950 (–)	4,666 (78.4%)	721 (12.1%)	563 (9.5%)
Age at incidence				
Mean (± SD)*	72.58 (± 10.17)	72.97 (± 10.21)	70.21 (± 10.04)	72.35 (± 9.65)
≤ 54	278 (4.7%)	213 (4.6%)	39 (5.4%)	26 (4.6%)
55–64	980 (16.5%)	740 (15.9%)	154 (21.4%)	86 (15.3%)
65–74	2,012 (33.8%)	1,509 (32.3%)	303 (42.0%)	200 (35.5%)
≥ 75	2,680 (45.0%)	2,204 (47.2%)	225 (31.2%)	251 (44.6%)
Marital status				
Married/common law	2,270 (38.2%)	1,642 (35.2%)	351 (48.7%)	277 (49.2%)
Single	500 (8.4%)	410 (8.8%)	32 (4.4%)	58 (10.3%)
Separated/divorced/widowed	576 (9.7%)	421 (9.0%)	85 (11.8%)	70 (12.4%)
Unspecified	2,604 (43.8%)	2,193 (47.0%)	253 (35.1%)	158 (28.1%)
Geographic area of residence				
Eastern	397 (6.7%)	316 (6.8%)	40 (5.5%)	41 (7.3%)
North Central	1,118 (18.8%)	844 (18.1%)	136 (18.9%)	138 (24.5%)
North West	1,795 (30.2%)	1,476 (31.6%)	100 (13.9%)	219 (38.9%)
South West	1,988 (33.4%)	1,388 (29.7%)	442 (61.3%)	158 (28.1%)
Tobago	652 (11.0%)	642 (13.8%)	3 (0.4%)	7 (1.2%)
Year of incidence**				
1995–1999	1,736 (29.2%)	1,351 (29.0%)	204 (28.3%)	181 (32.1%)
2000–2004	2,153 (36.2%)	1,716 (36.8%)	252 (35.0%)	185 (32.9%)
2005–2009	2,061 (34.6%)	1,599 (34.3%)	265 (36.8%)	197 (35.0%)
Method of detection				
Clinical presentation	1,929 (32.4%)	1,463 (31.4%)	268 (37.2%)	198 (35.2%)
Screening exam	609 (10.2%)	506 (10.8%)	62 (8.6%)	41 (7.3%)
Other/unknown/missing	3,412 (57.3%)	2697 (57.8%)	391 (54.2%)	324 (57.5%)
Stage at diagnosis				
In situ/localized	2,397 (40.3%)	1,817 (38.9%)	342 (47.4%)	238 (42.3%)
Regional	192 (3.2%)	146 (3.1%)	33 (4.6%)	13 (2.3%)
Distant	778 (13.1%)	590 (12.6%)	82 (11.4%)	106 (18.8%)
Unstaged	2,583 (43.4%)	2,113 (45.3%)	264 (36.6%)	206 (36.6%)
Grade				
Grade I	711 (11.9%)	529 (11.3%)	104 (14.4%)	78 (13.9%)
Grade II	1,059 (17.8%)	838 (18.0%)	143 (19.8%)	78 (13.9%)
Grade III	583 (9.8%)	449 (9.6%)	72 (10.0%)	62 (11.0%)
Grade IV	15 (0.3%)	10 (0.2%)	4 (0.6%)	1 (0.2%)
Unspecified	3,582 (60.2%)	2,840 (60.9%)	398 (55.2%)	344 (61.1%)
Morphology***				
Neoplasm, malignant	1,317 (22.1%)	1,076 (23.1%)	129 (17.9%)	112 (19.9%)
Carcinoma, NOS	317 (5.3%)	257 (5.5%)	33 (4.6%)	27 (4.8%)
Adenocarcinoma, NOS	4,244 (71.3%)	3,275 (70.2%)	550 (76.3%)	419 (74.4%)
Other/unknown/missing	72 (1.2%)	58 (1.2%)	9 (1.2%)	5 (0.9%)
Surgery				
Yes	1,287 (21.6%)	957 (20.5%)	191 (26.5%)	139 (24.7%)
No	2,639 (44.4%)	2,021 (43.3%)	328 (45.5%)	290 (51.5%)
Unknown/missing	2,024 (34.0%)	1,688 (36.2%)	202 (28.0%)	134 (23.8%)
Chemotherapy				
Yes	332 (5.6%)	224 (4.8%)	57 (7.9%)	51 (9.1%)
No	3,587 (60.3%)	2,751 (59.0%)	460 (63.8%)	376 (66.8%)
Unknown/missing	2,031 (34.1%)	1,691 (36.2%)	204 (28.3%)	136 (24.2%)
Radiotherapy				
Yes	582 (9.8%)	388 (8.3%)	108 (15.0%)	86 (15.3%)
No	2,671 (44.9%)	2,107 (45.2%)	310 (43.0%)	254 (45.1%)
Unknown/missing	2,697 (45.3%)	2,171 (46.5%)	303 (42.0%)	223 (39.6%)
Hormone therapy				
Yes	1,986 (33.4%)	1,460 (31.3%)	291 (40.4%)	235 (41.7%)
No	1,934 (32.5%)	1,516 (32.5%)	226 (31.3%)	192 (34.1%)
Unknown/missing	2,030 (34.1%)	1,690 (36.2%)	204 (28.3%)	136 (24.2%)
Immunotherapy				
Yes	8 (0.1%)	8 (0.2%)	0 (0.0%)	0 (0.0%)
No	3,908 (65.7%)	2,964 (63.5%)	518 (71.8%)	426 (75.7%)
Unknown/missing	2,034 (34.2%)	1,694 (36.3%)	203 (28.2%)	137 (24.3%)
Treatment received				
Yes	2,911 (48.9%)	2,133 (45.7%)	435 (60.3%)	343 (60.9%)
No/unknown	3,039 (51.1%)	2,533 (54.3%)	286 (39.7%)	220 (39.1%)
Vital status				
Mortality	3,423 (57.5%)	2,765 (59.3%)	357 (49.5%)	301 (53.5%)
Unknown	2,527 (42.5%)	1,901 (40.7%)	364 (50.5%)	262 (46.5%)

Percentages may not sum to 100% due to rounding

*Measures are not counts or percentages

All *p* values were ≤ 0.0001 except **p = 0.232, and ***p = 0.0209

Strikingly, the method of detection was missing in the dataset for most cases (57.3%), and only 10.2% of cases were initially detected at screening. Overall, a high proportion of the staged cases were at the localized stage (40.3%) followed by distant (13.1%) and regional (3.2%), with the highest frequency of CaP patients diagnosed with localized cancer observed among men of Indian ancestry (*p* < 0.0001). The majority of the cases had ‘unspecified’ tumor grade (60.2% overall). Across all ancestral groups, a significant proportion of the cases were diagnosed as Grade II with less than 1% diagnosed as Grade IV.

Overall, the morphology of the majority of CaP cases was adenocarcinoma, not otherwise specified (NOS) (71.3%). More than 30% of the treatment details were recorded as unknown, and in cases where treatment details were recorded, there were significant differences in the receipt of surgical treatment and initiation of chemotherapy, radiotherapy, hormone therapy, and immunotherapy by ancestry. Generally, only 45.7% of African ancestry patients received any treatment relative to 60.3% of Indians and 60.9% of mixed ancestry TT nationals.

**Fig. 2:**
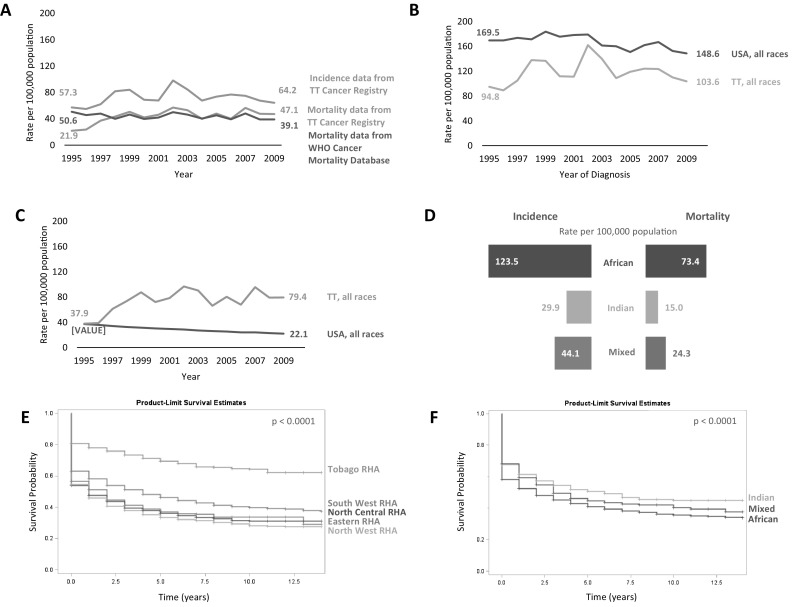
Prostate cancer rates, trends, and survival trends, Trinidad and Tobago, 1995–2009. **a** Trends in prostate cancer rates, Trinidad and Tobago, 1995–2009. Source: TT Cancer Registry and WHO Cancer Mortality Database. Rates are age adjusted to the 1960 world standard population. **b** Comparative prostate cancer trend analyses of Trinidad and Tobago and USA, 1995–2009: Incidence. Rates are age adjusted to the 2000 US standard population. **c** Comparative prostate cancer trend analyses of Trinidad and Tobago and USA, 1995–2009: Mortality. Rates are age adjusted to the 2000 US standard population. **d** Prostate cancer incidence and mortality rates in Trinidad and Tobago by ancestry, 1995–2009. Rates are age adjusted to the 1960 world standard population. **e** Prostate cancer survival probability by geography based on residence within Regional Health Authority catchment area. **f** Prostate cancer survival probability by ancestry

We also found that during the study period, there were 6,064 incident CaP cases for an effective rate of 64.2 per 100,000 and 3,704 deaths resulting in a mortality rate of 47.1 per 100,000 in 2009 (Fig. 2a). Given that the WHO provides global cancer mortality data, we compared the mortality rates calculated using National Cancer Registry of TT data to those reported in the WHO mortality database. The data showed that except for 2 years, WHO underestimated the rates from 1998 to 2009 (Fig. 2a). In fact, for 2009, the most recent year for which data were available from the National Cancer Registry of TT, the WHO underestimated the ASR mortality rate by 16.9%.

Several metrics of the registry data quality are provided in Supplementary Table 1. Of note, the percent of cases registered only on the basis of the death certificate (DCO) fluctuated from 16.55% in 1995 to 30.05% in 2000 to 17.91% in 2005 and then to 8.44% in 2009. The average over the entire period was 23.55%.

### Prostate cancer rates in TT relative to the USA

Next, we calculated the TT incidence and mortality ASR based on the 2000 US standard population to allow for comparisons to US CaP data. The US incidence rate decreased from 169.5 per 100,000 in 1995 to 148.6 per 100,000 in 2009, whereas in TT the rate increased from 94.8 per 100,000 to 103.6 per 100,000 over the same period (Fig. 2b). The mortality rate in TT increased between 1995 (37.9 per 100,000) and 2009 (79.4 per 100,000), while in the US the rate decreased from 37.3 per 100,000 to 22.1 per 100,000 over the same period (Fig. 2c).

### Prostate cancer incidence and mortality rates by ancestry

Given the demographics of TT, we examined CaP incidence and mortality rates (1960 world-standardized) by ancestry (Fig. 2d). Nationals of African ancestry had the highest incidence (123.5 per 100,000) and mortality (73.4 per 100,000), while those of Indian ancestry had the lowest incidence (29.9 per 100,000) and mortality (15.0 per 100,000).

### Prostate cancer survival rates

Unadjusted Kaplan–Meier curves show that CaP patients residing in the NWRHA catchment area had the lowest survival probability (5-year survival, 35%; 10-year survival, 30%) (Fig. 2a). For men in the North Central RHA (NCRHA) and Eastern RHA (ERHA), the 5-year survival rate was 37% and the 10-year survival rates were 30 and 32% (Fig. 2e). Interestingly, men in Tobago had the highest 5-year (70%) and 10-year (64%) survival probability. Unadjusted Kaplan–Meier curves showed that men of African ancestry with CaP had the lowest 5-year (42%) and 10-year (38%) survival probability, whereas men of Indian ancestry had the highest 5-year (50%) and 10-year (42%) survival probability (Fig. 2f).

### Prostate cancer mortality/incidence ratio

**Fig. 3:**
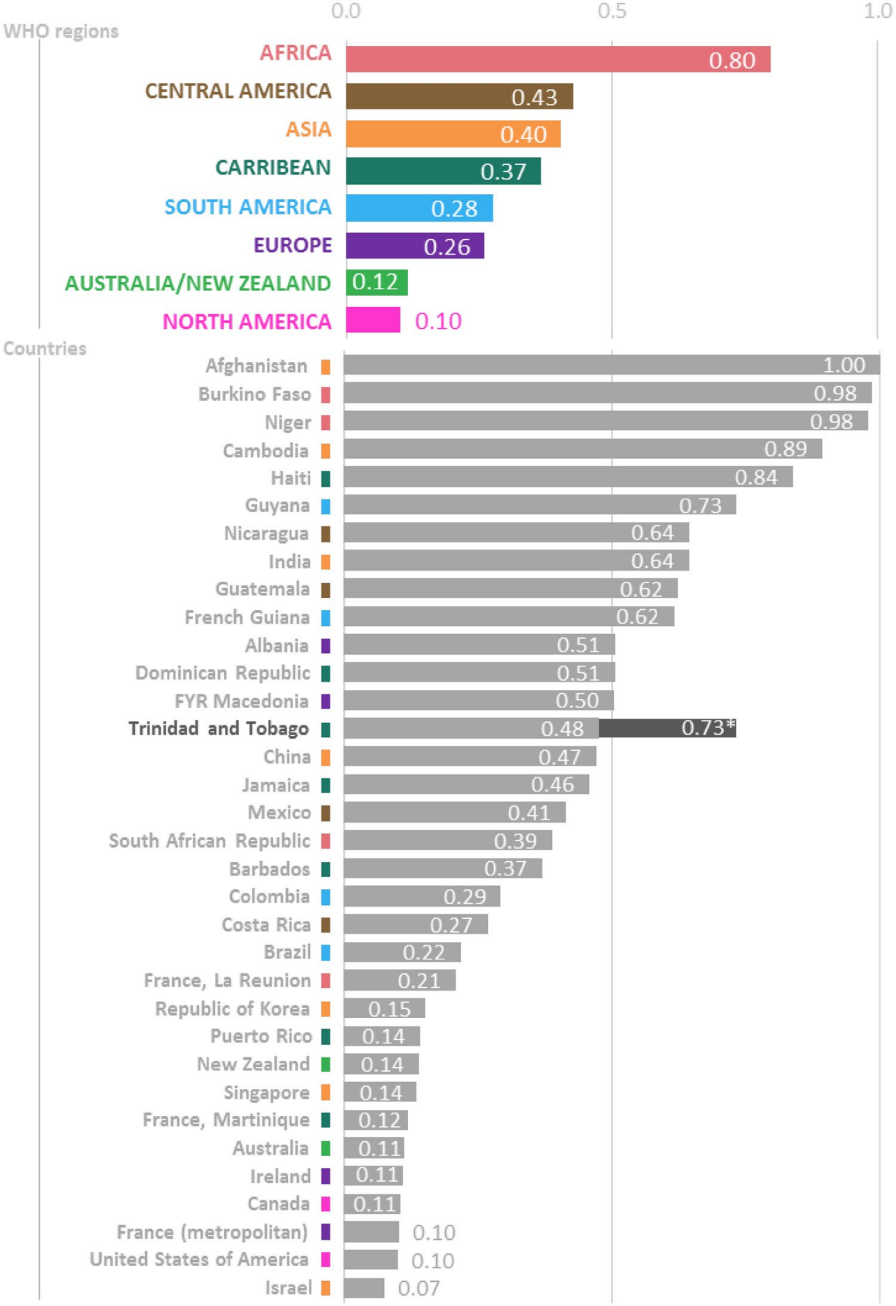
Prostate cancer mortality-to-incidence (M/I) rate ratio in World Health Organization (WHO) regions and select countries. *Data source* Incidence: Globocan; mortality: WHO Mortality database. *Data source Trinidad and Tobago Cancer Registry

The M/I ratio for TT was calculated using the National Cancer Registry of TT dataset for 1995–2009, while those for the WHO regions were calculated using incidence data from GLOBOCAN 2012 and mortality data from the WHO Mortality Database for 2012. In WHO regions, the M/I ratio ranged from 0.80 (Africa) to a low of 0.10 (North America) with a median value of 0.33 (Fig. 3). The TT M/I ratio was 0.48 which is higher than that of other countries in the Caribbean, such as Jamaica (0.46) and Barbados (0.37), but lower than that of Haiti (0.84) and Dominican Republic (0.51). TT had a lower M/I ratio than the highest ratio in all WHO regions except North America. Strikingly, when the National Cancer Registry of TT rates was used to calculate the M/I ratio, the TT ratio became 0.73 (rather than 0.48 as reported by WHO) bringing it on par with the M/I ratio of Guyana.

### Cancer incidence and mortality rates by geography

**Fig. 4:**
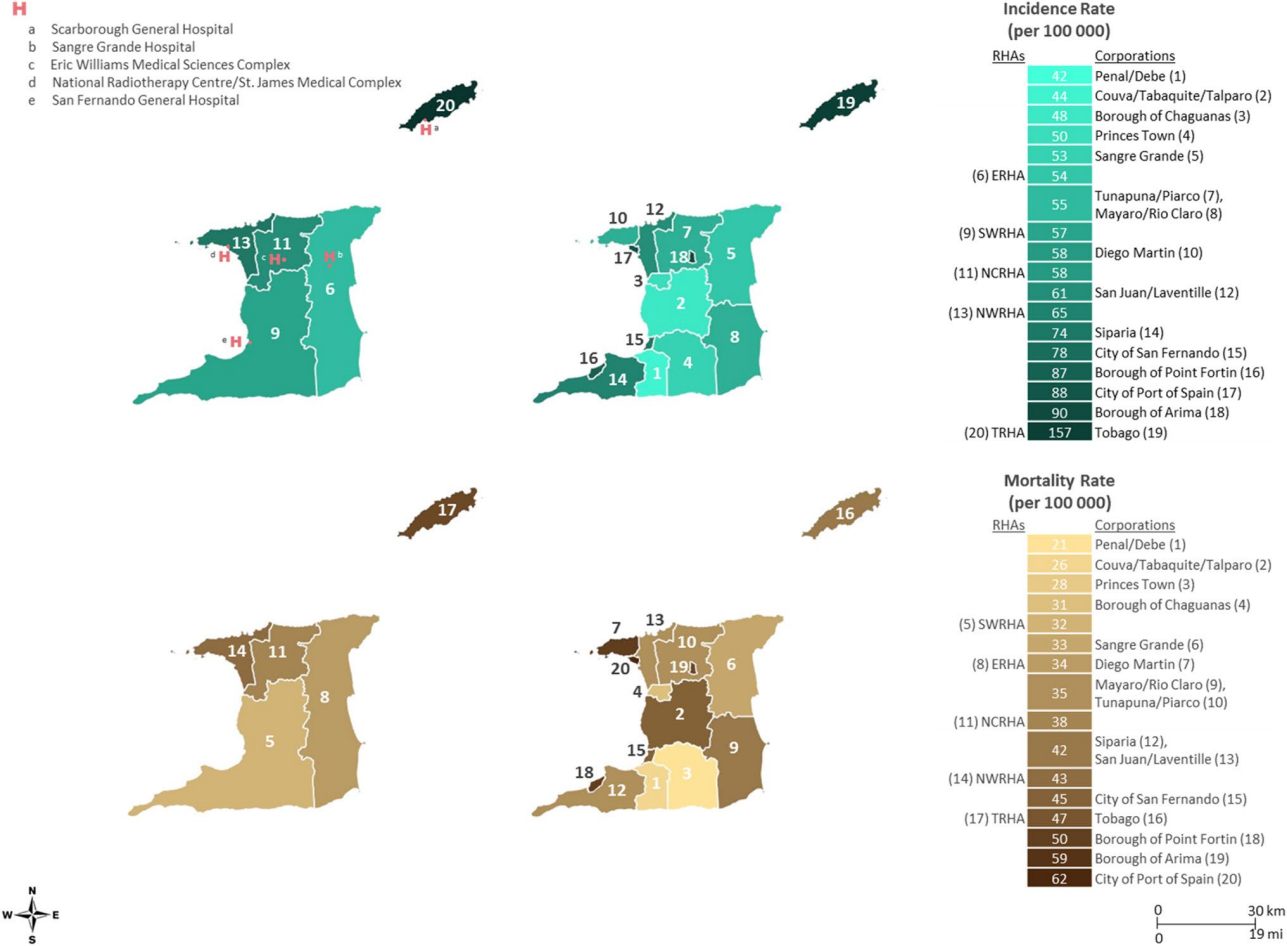
Geospatial maps of prostate cancer incidence and mortality rates in Trinidad and Tobago 1995–2009: top panel, left to right—age-standardized incidence rates for all Regional Health Authorities and Corporations, and bottom panel, left to right—age-standardized mortality rates for all Regional Health Authorities and Corporations. Rates are age adjusted to the 1960 world standard population

Geospatial mapping revealed associations between geography and TT CaP incidence and mortality rates for 1995–2009 (Fig. 4). In each RHA, there is at least one hospital providing varying levels of oncology services. There was significant variation in incidence rates (1960 world-standardized) between the Regional Health Authorities (RHAs) with the highest incidence rates observed in the Tobago RHA (TRHA) (156.5 per 100,000) and the lowest in ERHA (53.7 per 100,000) and the South West RHA (SWRHA) (57.0 per 100,000), respectively. The highest incidence rates occurred in Tobago (156.5 per 100,000), and four of the five corporations with the lowest rates were in the SWRHA. The highest CaP mortality rates (1960 world-standardized) (47.4 per 100,000) were reported in the TRHA with the lowest in SWRHA (32 per 100,000). The capital city of TT, Port of Spain, had the highest mortality (61.8 per 100,000) followed by the Borough of Arima (58.7 per 100,000). The five corporations with the lowest incidence rates also had the lowest mortality rates.

Table 2 reports the hazard ratios and confidence interval (CI) of CaP mortality by RHA. After adjusting for age, the mortality rates in the SWRHA (HR 0.80, 95% CI 0.74–0.87) and TRHA (HR 0.49, 95% CI 0.42–0.56) were significantly lower than observed in the North West (NWRHA). In multivariable adjusted models (adjusting for age at incidence, marital status, treatment initiation, stage, method of diagnosis), CaP mortality rates in the TRHA (HR 0.47, 95% CI 0.40–0.55) were significantly lower than observed in the NWRHA. We next examined differences in RHA mortality rates by ancestry (Table 3). For all RHAs, after adjusting for age, CaP patients of African ancestry (HR 1.13, 95% CI 1.01–1.26) had higher mortality rates compared to patients of Indian ancestry. In SWRHA, CaP patients of African ancestry (HR 1.23, 95% CI 1.05–1.43) had higher age-adjusted mortality rates compared to patients of Indian ancestry.

**Table 2:** Hazard ratios (HR) and 95% confidence intervals (CI) of mortality in each Regional Health Authority (RHA) catchment area in TT, 1995–2009

	NWRHA	ERHA	NCRHA	SWRHA	TRHA
Age adjusted	1.00 (ref)	0.943 (0.822, 1.081)	1.020 (0.929, 1.120)	**0.800 (0.737, 0.869)**	**0.486 (0.419, 0.562)**
Multivariable adjusted*	1.00 (ref)	0.900 (0.785, 1.032)	0.953 (0.867, 1.047)	0.956 (0.879, 1.040)	**0.469 (0.397, 0.553)**

Statistically significant (*p* < 0.05) estimates are bolded

*Multivariable models adjusted for age at incidence, marital status, detection method, cancer stage, and treatment (yes/no). The model was adjusted for initiation of any treatment

**Table 3:** Hazard ratios (HR) and 95% confidence intervals (CI) of mortality (B) in each Regional Health Authority (RHA) catchment area in TT, by ancestry, 1995–2009

	Ancestry		
	Indian	Mixed	African
All RHA			
Age adjusted	1.00 (ref)	1.008 (0.865, 1.176)	**1.129 (1.011, 1.261)**
Multivariable adjusted*	1.00 (ref)	1.030 (0.883, 1.202)	1.022 (0.914, 1.142)
Eastern RHA			
Age adjusted	1.00 (ref)	0.680 (0.367, 1.258)	1.046 (0.671, 1.631)
Multivariable adjusted*	1.00 (ref)	0.871 (0.467, 1.625)	0.998 (0.636, 1.567)
North Central RHA			
Age adjusted	1.00 (ref)	0.897 (0.657, 1.225)	1.012 (0.798, 1.283)
Multivariable adjusted*	1.00 (ref)	1.011 (0.738, 1.385)	1.007 (0.793, 1.279)
North West RHA			
Age adjusted	1.00 (ref)	0.986 (0.723, 1.346)	1.197 (0.919, 1.561)
Multivariable adjusted*	1.00 (ref)	1.055 (0.772, 1.441)	1.074 (0.823, 1.402)
South West RHA			
Age adjusted	1.00 (ref)	0.999 (0.765, 1.304)	**1.226 (1.047, 1.435)**
Multivariable adjusted*	1.00 (ref)	1.089 (0.833, 1.423)	1.144 (0.977, 1.341)
Tobago RHA			
Age adjusted	1.00 (ref)	0.301 (0.019, 4.811)	0.845 (0.118, 6.039)
Multivariable adjusted*	1.00 (ref)	0.077 (0.005, 1.308)	0.516 (0.069, 3.879)

Statistically significant (*p* < 0.05) estimates are bolded

^*^Multivariable models adjusted for age at incidence, marital status, detection method, cancer stage, and treatment (yes/no). The model was adjusted for initiation of any treatment

## Discussion

Our study is the first to analyze and comprehensively describe TT CAP incidence, mortality, and survival rates and trends from 1995 to 2009. Our findings indicated that the CaP burden in TT is high with rates that have been increasing over the study period. We identified CaP disparities by ancestry, geography, and age. The strength of our research lies in the analyses of all available cancer registry data from 1995 to 2009. Additionally, this is the first geospatial mapping of TT CaP incidence and mortality rates. Measuring and understanding the spatial and temporal distributions of the CaP burden according to geographic area in TT is critical for cancer prevention and control initiatives. Here we used a geospatial mapping approach that leverages CaP incidence and mortality data to identify disparities and areas that could benefit from targeted screening.

Our study indicates that the burden of CaP in TT in the population of African ancestry is higher than in the other ethnic groups. A screening program in Tobago which has been in existence for over a decade has identified a high prevalence of screening-detected prostate cancer among African Tobagonians compared to Asian Indian Tobagonians [23]. High CaP rates have been reported in black men across the African Diaspora including in the US [1, 24–26]. In the US, the age-standardized CaP mortality rate for black men is 48.2 compared with 9.7 for men overall [27]. The majority of the Barbados population share common West African heredity with black men in TT and the US albeit with low admixture rates [28]. In Barbados, the age-standardized (US) CaP mortality rate was 62.7 for the period 1995–2008 [29]. Interestingly, men in TT had an almost fourfold higher risk of death than US-born Black men in Brooklyn, whereas there was no significant difference in the risk of death for Caribbean-born black men in Brooklyn compared to US-born black men in the same area [30]. As we reported here for TT, it is possible that the CaP rates in the African diaspora are also underestimated [31]. This has implications for funding as well as cancer prevention and control efforts and therefore warrants further study.

The reasons for the higher CaP risk and burden as well as poorer outcomes among men of African ancestry are unclear. It is clear from our findings that men of African ancestry have the highest percentage of unstaged and the lowest percent diagnosed at a localized stage or Grade I. Additionally, we report that men of African ancestry are least likely to have received some form of treatment. These disparities are likely driven by deficiencies in the health care system, health-seeking behaviors and differences in the genomic background of CaP among different ancestries. Genetic studies have identified variants that confer increased CaP risk in people of African ancestry [32, 33]. Given the high CaP mortality rates in TT, there is need for genomic sequencing to identify clinically actionable genomic insults that can inform a CaP precision medicine framework [34]. Some studies have identified genomic alterations of prognostic clinical significance [35, 36]. Our study highlights the need to determine the genomic landscape of CaP in TT to ascertain whether ancestry-specific genetic insults might play a role in the disparities we report.

Given the higher rate of CaP among men of African ancestry that we report, it might be worthwhile to look at screening approaches in TT. Prior to 2001–2002, the PSA test was not readily available, increasing the risk that most men would present with advanced disease (unpublished data). In 2002, when it became available, there was reluctance by men in Trinidad to seek testing (unpublished data). This situation was different in Tobago where intensive, population-based, opportunistic screening efforts occurred [23, 25]. These studies reported a CaP prevalence about three to four times greater than rates reported from similar screening studies of predominantly Caucasian populations (ages 50–79 years) [25, 37–39]. Interestingly, in the same study, 12% of biopsied men aged 40–49 years were diagnosed with prostate cancer [25]. There is a need for the development of evidence-based screening guidelines specific to TT. The screening guidelines offered by United States Preventive Services Task Force [40], American Cancer Society [41], and the American Urological Association [42] are conflicting, derived from studies conducted with relatively few men of African ancestry.

A recent representative survey in TT reported that of 1,093 men only 28.6% and 46.6% of men in the 45–54 and 55–64 age groups, respectively, had a prostate examination [43]. This report might help to explain the fact that men in TT are diagnosed at more advanced ages than in several other countries. In TT, the mean age of diagnosis was 72.6 years, whereas a recent study reported 62 years for Americans, 66 years for Asian Indians, 69 years for Senegalese cases, and 68 years in Barbados [29, 44]. The advanced age of presentation suggests that the high percent of unstaged and ungraded cancers we report are likely those of men with advanced disease. Typically, men are less likely to present for medical advice, screening, and follow-up care even when faced with symptoms that deserve such attention [45, 46]. Layering this, in TT there are health-avoidant behaviors and attitudes such as the use of complementary and alternative medicine prior to seeking medical attention. In fact, 87% of respondents in a recent TT survey reported that herbal remedies had either equal or greater efficacy compared with conventional prescribed medicines [47]. This delay potentially results in patients presenting at a more advanced stage with a higher possibility of metastases. The reasons for the advanced age of presentation and the impact on clinical features are worthy of further study.

We report a high burden of CaP in TT along with gaps in the data on stage, grade, method of detection, and treatment. This suggests that there is need for more focused attention to ameliorate the trends we report. Given the high cost of treating CaP, there is need for stakeholders in the health sector to launch a national prostate awareness campaign to increase the awareness of prostate cancer, early detection, and prevention efforts aimed at modifiable risk factors such as smoking, proper exercise, and good nutrition.

There are several limitations inherent in this study. These results might reflect an underestimation of CaP rates as all cases are not systematically reported to the National Cancer Registry of TT as is required by law in the USA. Cancer surveillance via the population-based registry plays a critical function in formulating and monitoring the success of cancer control plans [48, 49]. Steps towards surveillance improvements in TT might include surveys and hospital chart reviews to quantify the level of under-notification as a first step towards improving the system. It is possible that some of the increase in prostate cancer mortality rates over the study period could be attributed to improvements in data quality by the registry. This possibility again highlights the need to improve cancer surveillance in TT. Additionally, neither the lack of information about measures of data quality by geographical area, nor case ascertainment by geographical area, makes any interpretation of the spatial patterns problematic. If reporting these geographical results in the abstract, it should be accompanied by a statement regarding the limited data quality and unknown impact on the variation. Further, a substantial number of cases were unstaged or had an unknown grade. Additionally, since ancestry was primarily self-reported or imputed in the absence of admixture analysis, the ancestry of all cases were not validated. The average DCO cases of 23.55% is slightly above the threshold set by IARC for inclusion in Cancer Incidence in Five Continents [50]. Taken together, these limitations can impact data quality and the interpretation of observed patterns and thus reflects a need for the TT cancer registry to improve data quality.

## Conclusion

Despite these limitations, the strength of this study lies in the analyses of all of the currently available CaP data collected from 1995 to 2009 in TT. We showed that there are associations by ancestry, geography, and age with respect to CaP incidence and mortality rates and trends in TT. Despite its status as a high-income country with universal healthcare access, the mortality rate in TT is one of the highest in the world. Our findings highlight the need for targeted screening and improved clinical approaches to ameliorate these high rates of CaP mortality. A key finding from this study is the urgent need for a more robust population-based cancer registry in Trinidad and Tobago as a first step towards reducing the burden we report.

## Electronic supplementary material

Below is the link to the electronic supplementary material.

Supplementary material 1 (XLSX 42 KB)
